# Leukemia Cutis Presenting as a Morbilliform Eruption: A Case Report and Literature Review

**DOI:** 10.7759/cureus.47018

**Published:** 2023-10-14

**Authors:** Fatimah M AlTassan, Tala A Qadoumi, Rama A Alhallaf, Fahad M Alsaif

**Affiliations:** 1 Department of Dermatology, King Fahad Medical City, Riyadh, SAU; 2 Department of Dermatology, King Khalid University Hospital, King Saud University, Riyadh, SAU

**Keywords:** case report, leukemia, dermatopathology, morbilliform eruption, leukemia cutis

## Abstract

Leukemia cutis (LC) is a broad term that describes the infiltration of neoplastic leukocytes into the skin. Classically, LC is characterized by erythematous papules and nodules. However, LC can have a widely variable presentation. Therefore, it is crucial to maintain a high index of suspicion for LC through a complete clinical assessment, histopathology, and immunohistochemistry to distinguish this entity from other clinical mimickers. Herein, we report a case of biopsy-proven LC presenting as a morbilliform eruption that was initially suspected to be a drug eruption in a child with acute monocytic leukemia.

## Introduction

Extramedullary manifestation of leukemia can occur in various organs of the body, including the skin. Leukemia cutis (LC), first described in 1913 by Reschad and Schilling-Torgau [1], is a broad term that describes the infiltration of neoplastic leukocytes or their precursors into the epidermis, the dermis, or the subcutis [2]. The onset of skin manifestation in relation to peripheral blood or bone marrow involvement is variable and often occurs concomitantly with, and sometimes, several months after the diagnosis of systemic leukemia. In rare occasions, LC may precede any evidence of bone marrow or peripheral blood involvement (so-called aleukemic LC) [2]. The reported frequency of LC ranges from 2% to 30% depending on the underlying form of leukemia [2]. LC can be seen with any type of leukemia. The most common cause of LC is adult T-cell leukemia, where the percentage of patients with LC is up to 70% [3]. The second most common cause of LC is acute myeloid leukemia (AML), especially with monocytic or myelomonocytic morphology, where the reported prevalence of skin involvement reaches up to 50% [3]. The frequency of LC is also higher among infants and children than adults [3]. LC most commonly presents as erythematous papules and nodules [2,4]. However, LC can have a widely variable presentation. Herein, we report a case of biopsy-proven LC presenting as a morbilliform eruption that was initially suspected to be a drug eruption in a child with acute monocytic leukemia. This case was previously presented as a conference poster at the 2022 annual King Saud University medical student council research forum.

## Case presentation

A 19-month-old girl was admitted through the emergency department to the oncology ward upon presentation with high-grade fever, generalized lymphadenopathy, and hepatosplenomegaly. Workup at presentation showed peripheral blood smear counts as follows: WBC: 3.8x10^9/L (normal range for patient’s age and gender: 6-18 x10^9/L), red blood cells: 3.06x10^12/L (normal range for patient’s age and gender: 3.5-5.2 x10^12/L), hemoglobin: 89 g/L (normal range for patient’s age and gender: 105-135 g/L), platelets: 37x10^9/L (normal range for patient’s age and gender: 140-450 x10^9/L), and 25% of leukocytes were immature along with marked neutropenia. Blood and urine cultures were negative. Unenhanced CT of the paranasal sinuses and intravenous contrast-enhanced CT chest, abdomen, and pelvis showed leukemic infiltration of the pancreas, kidneys, orbital floor, and paranasal sinuses and bone involvement (scapula). Bone marrow examination showed 80% blast which was of monocytic lineage upon immunophenotyping by flow cytometry. In addition, FISH signal patterns revealed chromosomal rearrangements, specifically t(9;11)(p21.3;q23.3); KMT2A-MLLT3. Therefore, she was admitted to the oncology ward with the diagnosis of acute monocytic leukemia with febrile neutropenia. The French-American-British (FAB) classification was AML-M5.

**Figure 1:**
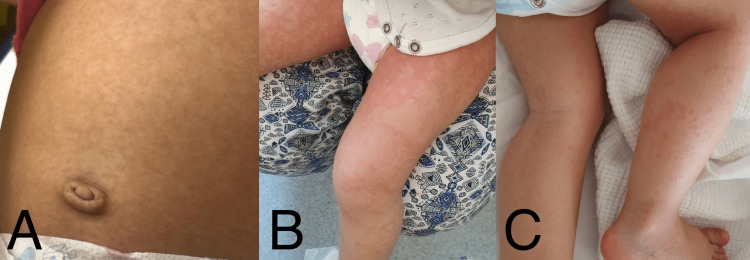
Multiple, edematous, erythematous macules and papules on the trunk (A) and lower extremities (B and C), with some coalescing into larger patches and plaques and some showing follicular accentuation

The dermatology team was consulted on day 10 of admission regarding pruritic skin lesions that appeared on day 6 of admission over the abdomen and upper extremities. The skin lesions then generalized to involve the lower extremities, hands, feet, and face and continued to increase in number. Systemic review was negative. As part of her management, she was started on multiple antibiotics by the oncology team. The mother also reported that the baby received a five-day antibiotic course before admission due to fever. At the onset of the eruption, she was not yet started on chemotherapy by the oncology team until infections were ruled out as she was still febrile. On physical examination, the patient was vitally stable. Vital signs were as follows: axillary temperature: 36.4 degrees Celsius, heart rate: 132 beats per minute, blood pressure: 107/63 mmHg, and oxygen saturation: 100% on room air. Facial edema with bilateral eyelid edema and erythema were noted. Multiple, edematous, erythematous macules and papules involving the face, upper and lower extremities, and trunk, with some coalescing into larger patches and plaques, and some showing follicular accentuation were noted (Figure 1A-1C).

The remainder of the skin and mucous membranes examination was unremarkable. Labs showed pancytopenia, eosinophils constituted 1.6% of WBC, and thrombocytopenia. The comprehensive metabolic panel was normal.

Our initial impression was a morbilliform drug eruption. Therefore, we recommended stopping the following culprit medications: piperacillin-tazobactam, amikacin, and vancomycin. We also recommended applying mometasone cream twice daily over pruritic lesions and 10 milligrams of oral loratadine daily.

**Figure 2:**
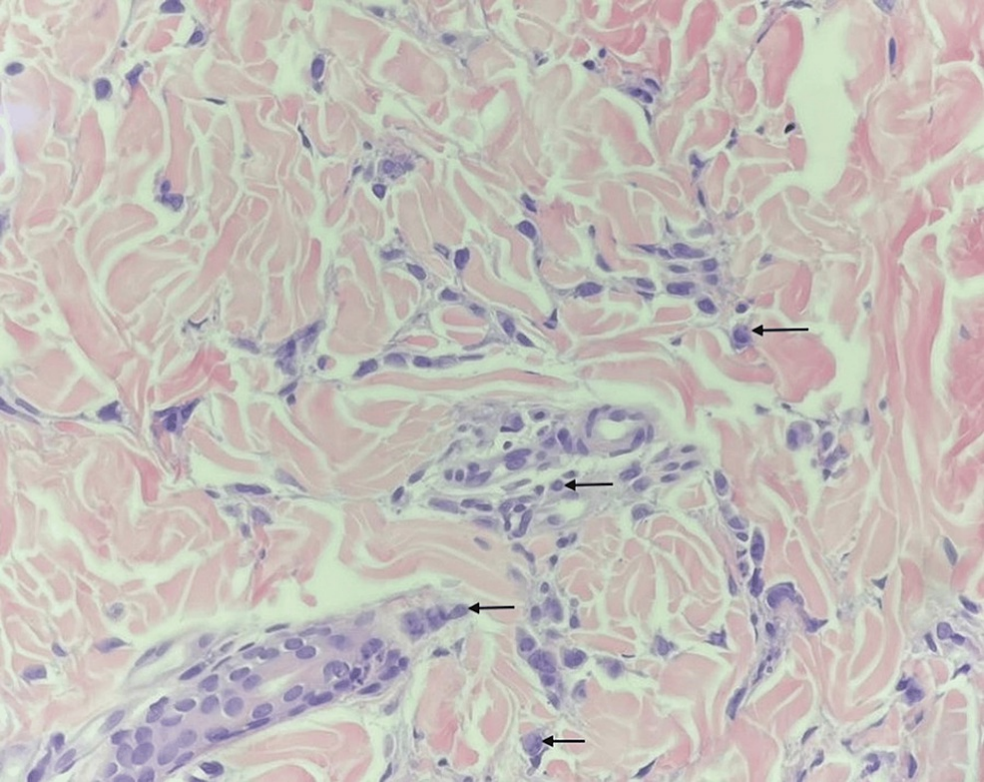
Punch biopsy demonstrating dermal interstitial and perivascular infiltrate composed of atypical hematolymphoid cells (arrows pointing to some of the cells) (hematoxylin and eosin x400)

Despite the management, the lesions continued to progress, as noted upon further follow-up. A 3-millimeter skin punch biopsy was then taken to rule out leukemic skin infiltration. Histopathologic examination showed atypical histiocytic dermal interstitial and perivascular infiltrate consistent with involvement by monocytic leukemia (Figure 2).

**Figure 3:**
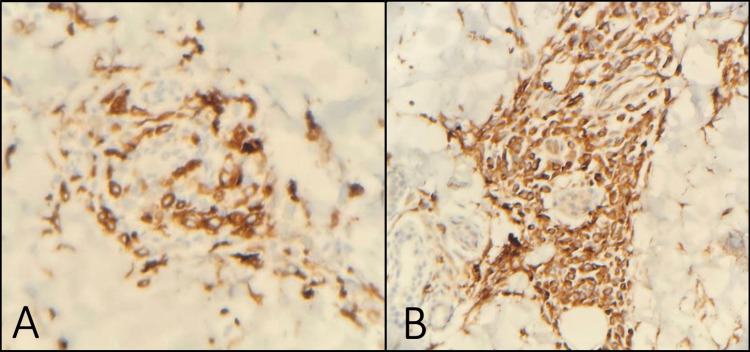
Immunohistochemical stains show the atypical cells are positive for CD68 (A) and CD163 (B)

On immunohistochemical stains, the cells were positive for CD68 and CD163 and negative for MPO, CD3, CD20, CD34, CD1a, S100, and CD117 (Figure 3). And a diagnosis of LC was made.

Intrathecal injection of cytarabine was given on day 9 of admission, and the first cycle of induction systemic chemotherapy was started on day 13 of admission. Skin lesions improved after induction chemotherapy with daunorubicin, cytarabine, and etoposide.

## Discussion

Cutaneous manifestations associated with leukemia are divided into specific and, the more frequently seen, non-specific manifestations [2]. Non-specific skin lesions, in which there is no leukemic infiltration into the skin, are either those that develop as a result of bone marrow failure or as a cutaneous paraneoplastic disease [2]. Inadequate hematopoiesis results in hemorrhagic skin lesions, secondary to underlying thrombocytopenia or coagulopathies, such as petechiae and purpura. Opportunistic infections can develop due to immunosuppression secondary to inadequate granulocytopoiesis or chemotherapy [2]. Reported paraneoplastic dermatologic manifestations include pyoderma gangrenosum, paraneoplastic pemphigus, Sweet syndrome [2,4], and insect-bite-like skin changes [5].

Specific skin lesions are those characterized by leukemic infiltration (LC) [2,4]. LC has a heterogeneous morphology, with no apparent site of predilection for skin involvement [4]. It has been described as solitary or multiple, generalized or localized, red-brown to violaceous papules, nodules, and plaques [4,6] or, in rare reports, as generalized asymptomatic erythematous maculopapular eruption [4,7,8]. The most common presentation is erythematous papules and nodules [2,4,6-8].

In a report of 42 cases with LC, the most frequent skin lesions, observed in 60% of the cases, were multiple papules and nodules. Twenty-six percent of the cases had infiltrative plaques and, less frequently, macules, noduloulcerative lesions, ulcers, bullous lesions, erythroderma, or oral lesions also were seen [4].

In contrast to our case, the lesions of LC are often asymptomatic. However, very few reports demonstrated pruritic lesions [6,9]. Cronin et al. [9] reported 33 cases of myeloid LC in which only one case had pruritic nodules and another case presented with generalized pruritic urticarial papules. Furthermore, in a report of 75 LC cases, Kang et al. [10] demonstrated only one case with pruritic erythematous papules on both extremities.

Kaddu et al. [7] described nine out of 26 cases that had LC presenting as generalized maculopapular eruption, clinically resembling a drug or viral eruption which is atypical for LC and adds difficulty to the diagnosis. The types of leukemia in these nine cases were acute myelomonocytic leukemia and chronic myelogenous leukemia.

Other unusual presentations of LC that have been reported were panniculitis resembling erythema nodosum [11], eczematous lesions [12], LC mimicking tumid lupus [13], acneiform eruption [6,9], infiltrative and crusted scalp lesions [9], annular plaques in the scalp [9], and blue dermal nodules [9].

Because the clinical findings of LC are variable, histopathologic and immunohistochemical evaluation is essential for diagnosis. Histological findings of myeloid LC are heterogeneous but typically characterized by a primarily interstitial infiltrate of leukemic cells in the dermis, sometimes extending into the subcutis, with sparing of the upper papillary dermis [4]. A perivascular and/or periadnexal pattern of involvement is also common [4].

The pathophysiology responsible for the migration of leukemic cells into the skin is not well understood. While some associations have been observed, no specific phenotype has been found to consistently lead to LC. The homing of T and B leukemic cells to specific tissues is controlled by the expression of different chemokine receptors and adhesion molecules. Expression of blast neural cell adhesion molecule (CD56), on leukemic blasts’ surface, has been implicated in extramedullary disease pathogenesis [14]. This CD56 molecule plays a role in cell-matrix adhesion, which may explain the observation that CD56 expression is significantly associated with skin involvement in patients with AML and other cutaneous lymphoma, compared to CD56-negative patients [14]. Furthermore, chromosome 8 abnormalities are more common in patients with AML with LC than in patients with AML without LC [14].

Our case’s immunohistochemical staining was positive for CD68 which is a marker found to be consistently expressed in skin-infiltrating leukocytes in LC (in 94-100%) and for CD163 which is highly specific for monocytic leukemia [9]. The immunohistochemical staining of myeloid LC in our case was negative for CD34 and CD117, which are principally used to define blasts in the bone marrow [9]. The absence of these markers in the skin is a typical finding in LC even when they are detected in the bone marrow flow cytometry [9]. Previous reports of immunohistochemical staining of myeloid LC were negative for CD34 and CD117 in the skin in the majority of cases even when bone marrow blasts expressed these antigens [9]. The demonstration of this discordance necessitates conjoining bone marrow and flow cytometry with skin findings to make the diagnosis of LC.

The presence of LC denotes a poor prognosis, systemic progression, and short survival in leukemic patients. Ninety percent of LC patients have extramedullary involvement of other body sites besides LC, with meningeal involvement being the most common extramedullary site, occurring in 40% of patients [15].

LC is a local manifestation of an underlying systemic disease; therefore, the management should be aimed at eradicating the systemic disease by using aggressive systemic chemotherapy. The treatment consists of intensive induction chemotherapy followed by either cytarabine-based consolidation or hematopoietic cell transplantation [16].

## Conclusions

LC is a rare cutaneous manifestation of common hematopoietic malignancies. In this report, we showed that LC can have a widely variable presentation by describing a rare case of biopsy-proven LC with a unique presentation as an extensive morbilliform eruption that was initially suspected to be a drug eruption in a child with AML-M5 FAB, with t(9;11)(p21.3;q23.3); KMT2A-MLLT3.

The relatively rare incidence of LC with its heterogeneous clinical presentation requires awareness among dermatologists, internists, and pediatricians, as these patients may initially present to these specialists. It is crucial to maintain a high index of clinical suspicion, through a complete history, physical examination, histopathology, and immunohistochemistry to distinguish this entity from other clinical mimickers.
